# Assessing the impact of the Good Samaritan Law in the state of Connecticut: a system dynamics approach

**DOI:** 10.1186/s12961-021-00807-w

**Published:** 2022-01-06

**Authors:** Nasim S. Sabounchi, Rebekah Heckmann, Gail D’Onofrio, Jennifer Walker, Robert Heimer

**Affiliations:** 1 Department of Health Policy and Management, Center for Systems and Community Design, City University of New York (CUNY) Graduate School of Public Health and Health Policy, 55 W. 125th Street, New York, NY 10027 United States of America; 2 Department of Emergency Medicine, Yale University School of Medicine, New Haven, CT United States of America; 3 Binghamton University-State University of New York, Binghamton, NY United States of America; 4 Department of Epidemiology of Microbial Diseases, Yale University School of Public Health, New Haven, CT United States of America

**Keywords:** Opioid use disorder, Emergency medicine, Health policy simulation, System dynamics modelling

## Abstract

**Background:**

Although Good Samaritan laws (GSLs) have been widely adopted throughout the United States, their efficacy in individual states is often unknown. This paper offers an approach for assessing the impact of GSLs and insight for policy-makers and public health officials who wish to know whether they should expect to see outcomes from similar policy interventions.

**Methods:**

Utilizing a system dynamics (SD) modeling approach, the research team conducted a policy evaluation to determine the impact of GSLs on opioid use disorder (OUD) in Connecticut and evaluated the GSL based upon the following health outcomes: (1) emergency department (ED) visits for overdose, (2) behavioral changes of bystanders, and (3) overdose deaths.

**Results:**

The simulation model suggests that Connecticut’s GSL has not yet affected overdose deaths but has resulted in bystander behavioral changes, such as increased 911 calls for overdose. ED visits have increased as the number of opioid users has increased.

**Conclusions:**

The simulation results indicate that the number of opioid-related deaths will continue to increase and that the GSL alone cannot effectively control the crisis. However, the SD approach that was used will allow policymakers to evaluate the effectiveness of the GSL over time using a simulation framework. This SD model demonstrates great potential by producing simulations that allow policymakers to assess multiple strategies for combating the opioid crisis and select optimal public health interventions.

**Supplementary Information:**

The online version contains supplementary material available at 10.1186/s12961-021-00807-w.

## Background and Introduction

The significant increase in the number of opioid overdose deaths in the United States over the past few decades is now widely recognized as a national public health crisis. Almost 11.4 million Americans aged 12 years or older misused opioids in 2017 [1], and more than 47, 000 people died from opioid overdoses in the same year [2]. As this death toll continues to rise, policy interventions become increasingly important as a means of reducing overdose deaths, and policymakers need tools to help guide decision-making. Most importantly, SD modeling is useful for studying resistance to public health interventions [3]. System dynamics (SD) modeling has gained momentum in the health sector due to its potential to address the challenges of decision-making for complex policy problems [4].

As part of the Centers for Disease Control and Prevention’s (CDC) Prescription Drug Overdose Prevention for States program, an SD approach was employed to evaluate the impact of Connecticut’s Good Samaritan law (GSL) by focusing on the following three health outcomes: (1) emergency department (ED) visits for drug overdose, (2) behavioral changes in bystanders, and (3) overdose deaths.

Opioid-related overdoses are now the leading cause of preventable death in the United States [5]. The magnitude of this public health problem is illustrated by the fact that the United States, with only 4% of the world’s population, accounts for 27% of the world’s opioid-involved deaths [6, 7]. In an effort to reduce the number of opioid-related deaths, almost all states have enacted some form of a GSL. These GSLs are intended to provide legal protection against liability and arrest for bystanders who give assistance during an overdose incident by either calling 911 or administering naloxone, in addition to protecting first responders and individuals who prescribe naloxone. Connecticut’s GSL was originally passed in 2011 and has been updated and expanded on a yearly basis since 2014 [8].

State-level ecological research has shown a 14–15% lower incidence of opioid overdose deaths in states with GSLs compared to those without these laws [9]. According to one study, GSLs are necessary in order to encourage help-seeking and lifesaving interventions in the event of an overdose; however, GSLs may be challenging to implement [10]. Moreover, while numerous studies have found that fear of police interactions [11, 12] has been the primary deterrent to people calling 911 during overdoses, many other factors have also been found to influence bystanders. For instance, some people fear that interactions with law enforcement might jeopardize their housing stability [12] or their employment [11]. People may also worry about having Child Protective Services contacted following an overdose with which law enforcement officers were involved [12].

In addition to fearing police interactions, a number of studies have found that lack of awareness about existing GSLs is one of the main factors limiting their impact [13]. Moreover, even people who know about GSLs are often still hesitant to call 911 because they are unsure about the specific protections afforded by the law. For example, some states require a review of an individual’s criminal background in order to determine eligibility for immunity [14]. Unfortunately, these details are often unknown in the midst of an overdose, leading to reluctance to call emergency medical services. This is a serious barrier to the full implementation of harm reduction policies because, according to one study, bystander participation is necessary during overdose events if help is to be summoned [15]. In fact, the results of another study showed that, for overdose events where bystanders had proper knowledge of the GSL, the likelihood of calling 911 was three times as high as in events where the bystander did not know about the GSL [16]. Thus, it might not be surprising that enacting the GSL in Connecticut has not yet resulted in a significant reduction in the number of opioid overdose deaths [17].

In order to better understand the rise in fatalities and the impact of the GSL in this complex environment, we applied an SD approach to account for the numerous factors that have moderated the impact of the GSL in Connecticut and to predict the future effectiveness of GSLs.

## Methods

According to Homer and Hirsch, “[a] system dynamics model consists of an interlocking set of differential and algebraic equations developed from a broad spectrum of relevant measured and experiential data” [18]. SD modeling is of particular importance to policymakers because it helps map out the components of health and prevention systems, explores their interactions, and identifies policy options that support the most efficient and effective arrangements of multiple elements within a system [3]. Recently, Homer and Wakeland [19] used an SD model to study the United States opioid epidemic and reflect upon the unintended consequences of intervention effects on opioid use disorder (OUD) and overdose deaths.

For the purposes of this analysis, we have developed and simulated an SD model using Vensim DSS software, version 8.2.1 [20]. The SD modeling approach incorporated measurement of multiple factors and their simultaneous variance in order to determine the effectiveness of the GSL in Connecticut. These factors include the number of ED visits for opioid drug overdose; the number of people using illicit drugs and misusing prescription drugs; the number of opioid-involved overdose deaths; and the behavioural changes in bystanders, including the number of police officers and members of the public who have GSL knowledge. While previous studies have used surveys, questionnaires, and participant interviews to allow researchers to evaluate the effectiveness of the GSL, SD modeling can provide a more structured understanding of the effectiveness of the GSL by describing the feedback loops and endogenous sources of system behavior that other modes of analysis are not designed to identify.

## The model

The model structure was developed and validated by involving several key stakeholders, including staff from the Connecticut Department of Public Health (CT DPH), researchers from Yale University, and members of local county health departments, during two participatory group model-building (GMB) sessions with the goal of developing a concept model that would serve as the focus for the rest of the SD modeling work. Participatory SD modeling was used to engage stakeholders in systems conceptualization and visual mapping of the dynamics that determine community-level opioid-related outcomes and to identify those dynamics that could be leveraged for systems improvement [21]. The concept model developed within the GMB sessions incorporated overdose deaths and behavioral change in bystanders to study the impact of the Connecticut GSL and served as an important transitional product that allowed us to incorporate other data sources and perform iterative simulations.

**Fig. 1:**
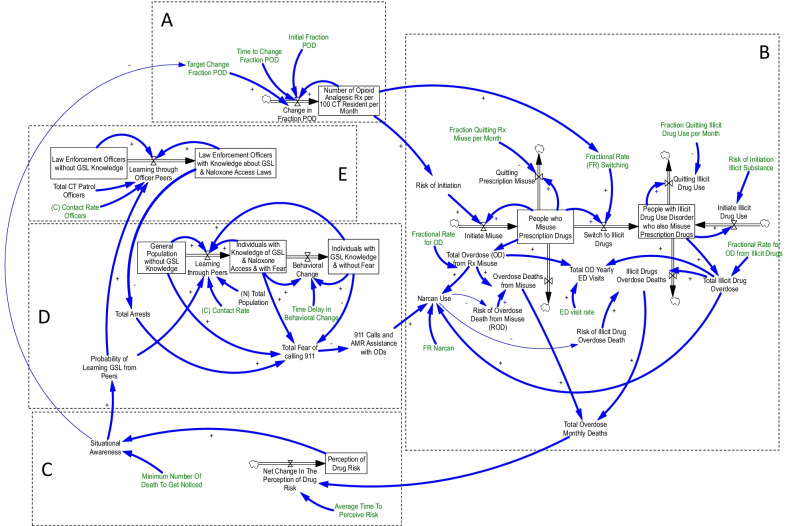
Simplified illustration of model

While many factors contribute to both prescription and illicit drug use, the change in the overall number of opioid drug prescriptions, as well as the rate of this change, certainly impacts the risk of initiation of drug misuse. Furthermore, illicit and prescription drug use are both affected by the amount of opioid prescribed. This is evidenced by several studies which have found that, while some policies lead to decreased OUD by reducing prescription supplies, other similar policies actually lead to an increased use of narco-trafficked drugs like heroin and fentanyl when individuals with OUD find alternative sources of opioids [22, 23]. In the model (Fig. 1), the assumption was made that, as *people who misuse prescription drugs* switch to illicit drugs, they would be counted as part of the *people with illicit drug use disorder who also misuse prescription drug*s group, which is consistent with the nomenclature and definition for illicit drug use as provided by the Substance Abuse and Mental Health Services Administration (SAMHSA) [24–26].

In Fig. 1, the model components are separated by dotted boundary lines. Located in the upper left portion of Fig. 1, section A depicts the part of the model that captures the change in the number of people being prescribed opioids over time (i.e. *number of opioid analgesic* *Rx per 100 Connecticut residents* *per month*). In a manner similar to the sharp increase in the *number of opioid analgesic* *Rx* provided in the mid- to late 1990s that contributed to a significant increase in the number of *people who misuse prescription drugs* [27], the change in “*number of opioid analgesic* *Rx* influences section B, located on the right-hand side of the model, which depicts the number of *people who misuse prescription drug*s and the number of *people with illicit drug use disorder who also misuse prescription drugs*. However, despite the overlap between prescription opioid use and illicit drug use, most patients with OUD do not necessarily become addicted from starting with prescription opioids. Moreover, analysis of opioid overdose data performed by the CDC’s Injury Center shows that the second wave of overdose deaths in 2010 involved heroin use, while the third wave, which started in 2013, involved synthetic opioids such as illicitly manufactured fentanyl [28].

Thus, this portion of the model illustrates the number of people who initiate either prescribed or illicit drug use, the number of people who transition from prescription to illicit drug use, the number of people who quit either type of use by getting into drug treatment programs, and finally, the number of people who die from overdoses.

While incorporation of these elements into the SD framework is crucial for its proper functioning, this analysis is most concerned with the portions of the model that reflect the effectiveness of the GSL in reducing overdose deaths. Located at the bottom of Fig. 1, section C indicates that the *net change in the perception of drug risk* is influenced by the number of opioid overdose deaths. Community members take notice of overdoses and may begin thinking about ways to prevent overdose deaths. The perceived risk of drug use, in conjunction with the parameter value representing the length of time over which this perception develops (Table 1), impacts the *situational* *awareness* of opioid use. In this way, the model demonstrates how an individual’s knowledge of the GSL is either unaffected or improved over time.

**Table 1:** Parameter values

Parameter (definition and unit of analysis)	Value in the model	95% confidence interval
Average time to perceive risk (months)	13.1131	12–17.7579
Risk of initiation of illicit substance (fraction of susceptible users initiating illicit drug use per month)	0.0024	0.0019–0.0028
ED visit rate (average number of times that people who misuse Rx or with illicit drug use disorder visit ED)	1.37808	1.2096–1.5465
Fraction quitting Rx misuse (fraction of users misusing Rx who quit per month)	0.002	0.0015–0.002
Fraction quitting illicit drug use (fraction of users using illicit drugs/misusing Rx who quit per month)	0.0007	0.0003–0.0012
Fractional rate for overdose among nonmedical users of Rx drugs (users per month)	0.0011	0.0011–0.0011
Fractional rate for overdose from illicit drugs and Rx misuse (users per month)	0.0037	0.0037–0.0037
Risk of overdose death from Rx misuse (fraction of Rx misuse OD incidence that leads to death)	0.00001	0.00001–0.0126548
Risk of overdose death from illicit drug use and Rx misuse (fraction of illicit drug OD incidence that leads to death)	0.2072	0.2021–0.2125

*Situational awareness* has a clear impact on changes in behaviour and, therefore, is directly connected to the left-hand side of the model (sections D and E). In other words, as the number of people dying from opioid overdose changes, the perception of the risk of drug use influences awareness in a region, determining how much law enforcement staff and the public learn about the GSL through peer interactions (i.e. *probability of learning GSL from peers*). Through these interactions, section E captures the changes in the number of *law enforcement officers with GSL knowledge*, in addition to capturing a corresponding change in the number of drug-related arrests. Conversely, the number of arrests can influence the willingness of bystanders to contact law enforcement for help in the event of an overdose. In this way, the model demonstrates the means through which people in Connecticut are either more or less inclined to call 911 and take advantage of the protection afforded by the GSL.

The interaction between fear of police interactions and knowledge of the GSL is incorporated into the center of the model in section D, which contains variables for the *general population without GSL knowledge*, *individuals with knowledge of the GSL and naloxone access and* *with fear* of calling 911, and *individuals with GSL knowledge and naloxone access and without fear* of calling 911. Just as police officers are made more or less aware of the risks of opioid use, harm reduction policies, and naloxone access through changes in situational awareness, the general population’s knowledge is also affected. Furthermore, the rate at which members of the general public and police officers learn about GSLs is also dependent upon the contact rate in a given region, meaning that more interactions throughout the day with people with knowledge of the GSL may result in more people learning about the GSL. The parameter value that addresses contact rates can be adjusted to reflect the population density in a region. Section D also shows that, while knowledge of the GSL may increase quickly, it takes time to quell the fear of calling 911 and, thereby, modify bystander behaviour. However, once fear drops, the number of 911 calls and emergency medical services staff arriving at overdose events will increase. Section D of the model demonstrates that, as people are more or less afraid of calling 911 during opioid overdoses, the number of 911 calls decreases or increases, respectively, thereby impacting the number of overdoses during which naloxone is administered, with a differential impact on mortality appreciated. The detailed model formulations are provided in the online supplementary information  (Additional file 1).

## Data and model calibration

Since most of the parameters defined in the model were not available in the relevant literature, model calibration was used to make estimates for the parameter values shown in green in Fig. 1. Constraints on plausible values of the calibrated parameters listed in Table 1 were formulated from expert opinion and the literature [29–32]. A full list of calibrated parameter values is provided in the online supplementary information (the Additional file 1). Calibration was performed using Vensim DSS, version 8.2.1 [20]. The calibration module in Vensim modeling software calculates the optimum values of model parameters using a maximum likelihood estimation approach [33] to create simulations which align best with real-world data, replicate historical trends, and create estimations for the future.

The data used to calibrate this model were collected from numerous sources over different intervals and describe the status of opioid use in Connecticut from 2009 to 2018. Information about overdose deaths was collected from the Connecticut Office of the Chief Medical Examiner (OCME). The data concerning the number of ED visits for overdose came from Connecticut Hospital Association (CHIME) discharge data. The researchers also utilized information from www.CTData.org, including the amount of illicit drug use other than marijuana between 2008 and 2014 (collected by SAMHSA as part of the National Survey on Drug Use and Health [NSDUH]) [34] and the rate of arrests due to drug law offences from 2010 to 2016 [35]. The most recent SAMHSA reports from 2015 to 2018 provide information about illicit drug use and prescription drug misuse in Connecticut, and this information was used to validate the simulation results [24–26]. Specifically, the illicit drug use described in the SAMHSA report includes the misuse of prescription psychotherapeutics, and the research team used this information to validate the total sum of *people who misuse prescription drugs* and the number of *people with illicit drug use disorder who also misuse prescription drugs* in the model.

The rate of opioid prescriptions per 100 Connecticut residents was retrieved from the CDC report on United States state prescribing rates [36]. Additionally, information concerning the rate of administration and dose of naloxone used was supplied by the American Medical Response (AMR) transportation company, which serves a large portion of the state of Connecticut. Lastly, the information on knowledge of the GSL and fear of calling 911 was provided by the following two survey reports: (1) the CT DPH and Central Connecticut State University’s (CCSU) survey on basic understanding of the GSL and the corresponding fear of calling 911 [37] and (2) the High Intensity Drug Trafficking Area’s (HIDTA) Heroin Response Strategy project report on the GSL’s impact on Connecticut policing practices [38].

## Modelling and simulation results

SD modeling has allowed the research team to capture the complex interrelationships among several key health outcome measures that drive the opioid epidemic in Connecticut. These outcomes include ED visits due to overdose, behavioral changes in bystanders, changes in perception of the risk of drug use, awareness of harm reduction policies, and overdose deaths.

**Fig. 2:**
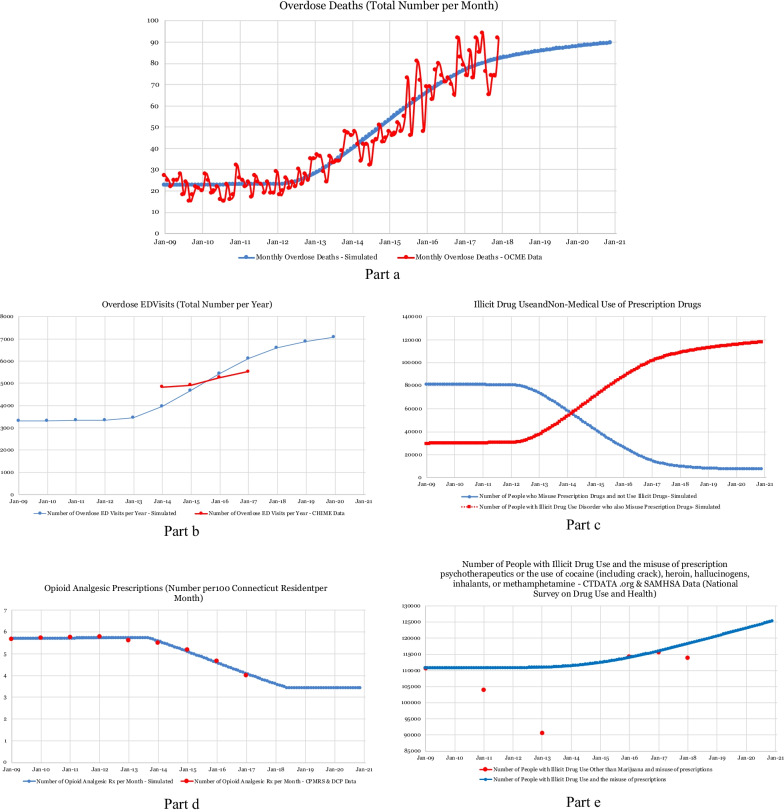
Simulation results for overdose deaths and drug use

First, the simulated number of overdose deaths aligns very closely with data supplied by the OCME. Unfortunately, these results reveal that the number of overdose deaths has been increasing over the past 8 years and will continue to grow if no additional action is taken (Fig. 2a). Additionally, the simulated number of ED visits for overdose aligns with the data supplied by CHIME discharge data, which shows that as the number of opioid users and overdose incidents increases, ED visits also increase (Fig. 2b).

Secondly, the model demonstrates that, as more opioids are prescribed, the use of illicit and prescription drugs increases. The simulation indicates that, as more people misuse prescription drugs, an increased number of people switch from prescribed to illegal opioids. For this reason, the number of people who misuse prescription drugs but do not use illicit drugs was shown to decrease around 2013; and the number of people who use illicit drugs and also misuse prescription drugs was shown to increase during the same time period (Fig. 2c). It is interesting to note that, around 2013, the rate at which opioid prescriptions were being written in Connecticut was decreasing (Fig. 2d). Consequently, this model supports the hypothesis that an increase in deaths and subsequent *situational awareness* could lead to decreased opioid prescriptions; and the corresponding link depicted in the model has been shown, thus far, to be an effective means of harm reduction (Fig. 1).

However, there is a mismatch between simulation results and the data points corresponding to illicit drug use and misuse of prescription drugs prior to 2014 (Fig. 2e). The simulation results show that the total number of people who use illicit drugs and/or misuse prescription drugs (i.e., the sum of *people who misuse prescription drugs* and the number of *people with illicit drug use disorder who also misuse prescription drugs* in the model) has increased overall, but data show a decrease between 2009 and 2013. One explanation is that, although the data sources for illicit drug use are all based on SAMHSA reports, the SAMHSA NSDUH reports introduced an independent multistage area probability sample as the first level of stratification in 2014 within each state. Thus, the data points between 2015 and 2018 are based on an updated data collection process; and there are likely inconsistencies between data collection methods before and after 2014. However, the SAMHSA reports were the most reliable data source available for our modeling purposes and, hence, were utilized in this study.

**Fig. 3:**
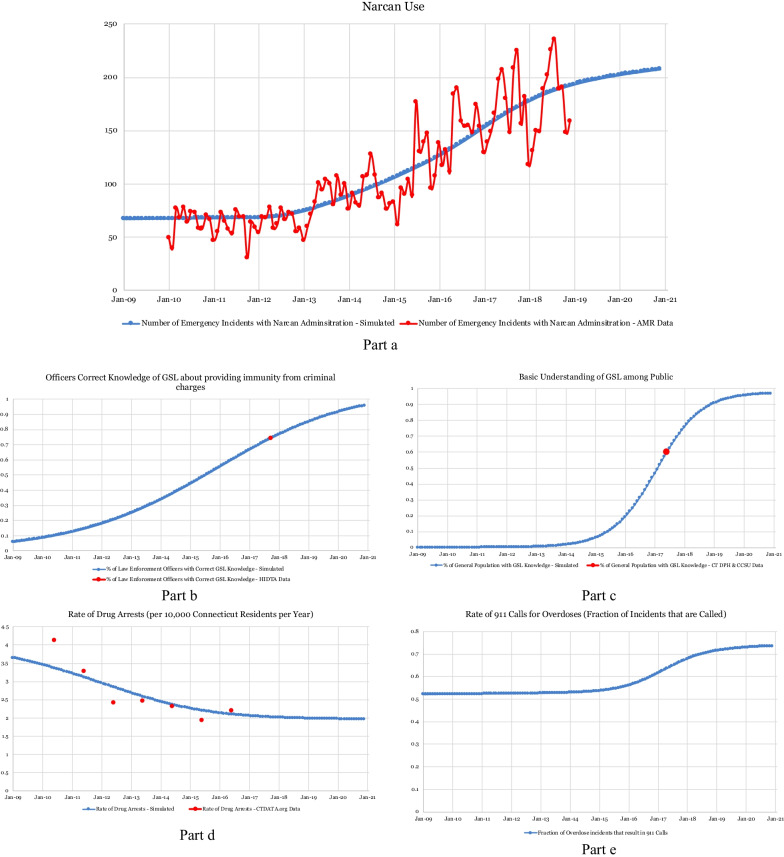
Simulation results for behavioral changes

Also, the model predicts that GSL knowledge will continue to grow from the 2017 estimates that indicate that 60% of the general public and 74% of police officers in Connecticut knew about the GSL [37, 38] (Fig. 3b, c). This is a prediction based on the model structure and estimates, not related to a specific educational plan. As shown in Fig. 1, the model structure shows a positive link from *perception of drug risk* to *situational awareness for drug risk*. A positive link describes a causal relationship in an SD model when the cause and effect change in the same direction. As more people perceive the high risk of drug use, they become more situationally aware. As a consequence, the *probability of learning about the GSL from peers* increases, which contributes to an increased number of law enforcement officers and members of the general public who learn about the law. We calibrated the model to identify the strength of these causal links by estimating the parameter values highlighted in green in Fig. 1, including the average time that it takes for GSL knowledge to change and the threshold number of overdose fatalities at which changes in awareness towards the risk of drug use and the benefit of GSLs will occur.

Policy makers may infer from the model that, because the number of overdose deaths is increasing, situational awareness will change, leading to an increase in officer and public awareness of the GSL. Subsequently, the number of people with GSL knowledge and without fear of calling 911 is anticipated to increase. Also, the rate of drug arrests per 10,000 for drug law offences has been decreasing, possibly due to the growth of GSL awareness, but is predicted to remain stable in the future (Fig. 3d).

Finally, the results of the simulation that represent the use of naloxone correspond to the data received from AMR, which show an overall increase in the number of overdose events during which naloxone is administered (Fig. 3a). The model explains that this increase is partially due to an increase in overdose incidents since 2009 and partially due to a similar increase in the number of 911 calls since the start of the simulation (Fig. 3e). However, even accounting for an increase in emergency medical assistance and administration of naloxone, the number of overdose deaths has continued to increase (Fig. 2a); and risk of overdose remains stable (Table 1), contrary to our initial hypothesis that had assumed negative causal links from *Narcan use* to *risk of overdose death from misuse (ROD)* and *risk of illicit drug overdose death* in Fig. 1. The increase seen over time in the dose of naloxone administered for an overdose from an average dose of 1.18 mg in 2010 to 1.94 mg in 2018 is based on data received from AMR and may be related to the high potency of the new illicit drugs that have become available over the past few years.

## Discussion

SD modeling is an analytical tool that helps policy makers approach difficult decisions in the presence of the uncertainties that complex problems create [3]. The SD approach is ideal for evaluating the delayed impact of the GSL on behavioral changes because it allows researchers to investigate the long-term effects of policy interventions using a simulation framework. Because of the ability to simulate with SD, policy makers can infer that, although the evidence currently demonstrates mixed effects of the GSL, the overall amount of drug use and number of overdose deaths will increase if no additional policies are implemented.

Moreover, because the model’s simulation results align with real-world data and can be used to replicate historical trends, it is reasonable to infer that the model is situationally relevant and may be used to evaluate what-if simulation scenarios. Policy makers may use this model to test new interventions that might be used to address the opioid crisis. Additionally, once more robust data on the behavioral impact of the GSL become available, those data can be used to produce an even more reliable model in the future.

While existing data show that the GSL has not yet reduced the number of overdose deaths, the model’s simulations indicate that the high number of deaths will likely foster an increased awareness of the GSL, leading to decreased fear of calling 911 and increased naloxone administration. However, the model also suggests that the overall trend of increased deaths may continue to grow despite this increased awareness. This prediction is supported by the model, as well as by many other studies, indicating that fear of police interactions is the primary reason that bystanders do not call 911.

Fortunately, the model demonstrates that interventions like the GSL, which protects bystanders against liability for providing assistance during overdoses, may represent a partial solution to this problem. However, additional interventions are needed to improve the effectiveness of the GSL. For example, although the model’s results indicate that the rate of drug-related arrest has slowed in recent years, future interventions, such as increased training for police officers, may still be necessary. One study claims that GSLs and other harm reduction policies are necessary but insufficient, primarily due to problems with implementation and awareness [10].

While efforts to alter the course of the opioid epidemic will require ongoing research concerning the numerous interventions that could be applied to this problem, this analysis illustrates how SD modeling may be beneficial in aiding policy makers who are tasked with decision-making in the setting of complex challenges. Because more time must pass in order to observe the long-term effectiveness of the GSL, an SD modeling approach can be used to make predictions about its long-term impact by employing a simulation framework. Additionally, since the model structure and feedback loops are relevant to the opioid epidemic in general, the model parameters can be calibrated towards historical data trends for other geographical regions or states and, thus, be customized for different locations and settings. In this way, policy makers can utilize this model to test future trends and determine the best solutions for various public health problems.

## Limitations

This SD model included feedback processes and dynamics important to understanding the impact of GSLs and providing insight for policy makers and public health officials. While this model provides a useful foundation for answering targeted research questions about GSLs and related policies, our analysis had several limitations. First, identifying relevant existing data sources for some model indicators that span the time horizon of the study has been challenging. Also, some data sources, such as the SAMHSA reports [24–26], have updated their data collection process, which has likely led to inconsistencies in data collection methods. While the current model structure and feedback loops appear to replicate historical trends well, future iterations of the model may test the alignment of current or additional feedback loops. Additionally, the model was designed based upon expert input and GMB sessions conducted with key stakeholders. A next step could adopt a more inclusive approach, and other relevant stakeholders such as patients, providers, law enforcement officers, and first responders could be invited to contribute to the modeling process and corresponding validation while exploring specific questions related to the impact of opioid policies on reducing overdose risk and fatality.

## Conclusions

SD modeling has been proven to be a useful approach for assessing the effectiveness of public health policy interventions through its utilization of a simulation framework. While other analytical methods may require research involving study participants and clinical trials, SD modeling allows for the prediction of the future effectiveness of interventions through its ability to replicate historical trends. While investigating the impact of the GSL on overdose deaths, ED visits and bystander behavior in Connecticut is the main purpose of this analysis, this model has also demonstrated great potential by producing simulations that reveal multiple strategies to aid policy makers in determining the best public health interventions for combating the opioid crisis.

## Supplementary Information

**Additional file 1.** **Section 1:** Model Formulation. **Section 2:** Estimation and Calibration of Model Parameters.

## Data Availability

All data generated or analyzed during this study are included in this published article and its additional information file.

## Abbreviations

GSL: Good Samaritan law
SD: System dynamics
SAMHSA: Substance Abuse and Mental Health Services Administration
ED: Emergency department
OUD: Opioid use disorder
CDC: Centers for Disease Control and Prevention
AMR: American Medical Response
GMB: Group model-building
