# Supplementary material for: Assessing the impact of the Good Samaritan Law in the state of Connecticut: a system dynamics approach
Source: Health Res Policy Syst. 2022 Jan 6;20:5. doi: 10.1186/s12961-021-00807-w (PMC8734429; doi:10.1186/s12961-021-00807-w)
Supplement: Supplementary file 1 — Additional file 1. Section 1: Model Formulation. Section 2: Estimation and Calibration of Model Parameters. [file 12961_2021_807_MOESM1_ESM.docx]

**Section 1: Model Formulation**

In this section, the model formulation is presented in the similar format as Vensim software. The main equations for each subsection of the model, are provided under the corresponding section.

**Simulation Set up**

1. INITIAL TIME = 0 (Represents the beginning of 2009)

**Unit**: Month

~ The initial time for the simulation.

1. FINAL TIME = 143 (Represents the end of 2020)

**Unit**: Month

~ The final time for the simulation.

1. SAVEPER = TIME STEP

**Unit**: Month

~ The frequency with which output is stored.

1. TIME STEP = 0.03125

**Unit**: Month

~ The time step for the simulation.

**Main Equations**

**Section A:**

1. Number of Opioid Analgesic Rx per 100 CT Resident per Month=

INTEG (Increase in Opioid Rx Rate-Decrease in Opioid Rx Rate, Initial Opioid Rx Rate)

**Unit**: 1/Month

1. Decrease in Opioid Rx Rate=

PULSE( start to switch2 , duration switch )*(-Target Change Opioid Rx Rate+ Number of Opioid Analgesic Rx per 100 CT Resident per Month )/(Time to Change Rx Opioid Rate/2)

**Unit**: 1/(Month*Month)

1. Increase in Opioid Rx Rate=

IF THEN ELSE (Time>=start to switch2 , 0 , 1 )*Number of Opioid Analgesic Rx per 100 CT Resident per Month*Fraction Opioid Rx Rate

**Unit**: 1/Month/Month

1. Target Change Opioid Rx Rate=

((Max Opioid Rx Rate-Min Opioid Rx Rate) * EXP(-Situational Awareness/Beta Coefficient2) + Min Opioid Rx Rate )

**Unit**: Dmnl

1. Max Opioid Rx Rate=

5.755

**Unit**: Dmnl

1. Initial Opioid Rx Rate=

5.675

**Unit**: 1/Month

~ Rx Opioid Prescribing Rate per 100 Persons in 2009 from CDC reports

**Section B:**

1. Initial Number of Illicit Drugs Users=

Total Users*(1-FR NMUOP)

**Unit**: People

1. Initial Number of People Misusing Rx Drugs=

Total Users*FR NMUOP

**Unit**: People

1. Risk of Overdose Death (ROD)=

Target ROD from Rx Misuse

**Unit**: Dmnl

1. Total Illicit Drug Use=

People who Use Illicit Drugs or Misuse Prescription Drugs + People who Misuse Prescription Drugs

**Unit**: People

1. Initiate Illicit Drugs=

Risk of Illicit Drug Use Initiation *(People who Use Illicit Drugs or Misuse Prescription Drugs)*Susceptible/"(N) Total Population"

**Unit**: People/Month

1. Risk of Overdose Death from Illicit Drug=

Target ROD from Illicit Drug

**Unit**: 1/Month

1. People who Use Illicit Drugs or Misuse Prescription Drugs =

INTEG (Initiate Illicit Drugs+ Switch to Illicit Drugs-Illicit Drugs Overdose Deaths-Quitting Illicit Drug Use, Initial Number of Illicit Drugs Users)

**Unit**: People

1. People who Misuse Prescription Drugs=

INTEG (Initiate Misuse-Overdose Deaths from Misuse-Quitting Prescription Misuse-Switch to Illicit Drugs, Initial Number of People Misusing Rx Drugs)

**Unit**: People

1. Total OD ED Visits=

INTEG (ED visit rate*(Total OD+Total OD Illicit)-Total OD ED Visits*switch year/TIME STEP,ED visit rate*(Total OD+Total OD Illicit)*12)

**Unit**: People

1. Illicit Drugs Overdose Deaths=

Risk of Other Overdose Death*Total OD Illicit

**Unit**: People/Month

1. Overdose Deaths from Misuse=

"Risk of Overdose Death (ROD)"*Total OD

**Unit**: People/Month

1. Initial Overdose Deaths=

INITIAL(Initial Number of People Misusing Rx Drugs *Risk of Overdose Death (ROD)*FR OD + Initial Number of Illicit Drugs Users *Risk of Other Overdose Death*FR OD Illicit)

**Unit**: People/Month

1. Monthly Death Rate from Illicit Drug Overdose=

INTEG (Illicit Drugs Overdose Deaths-Drop out Illicit Drug Deaths from Previous Month, Initial Number of Illicit Drugs Users *Risk of Other Overdose Death*FR OD Illicit)

**Unit**: People

1. Monthly Death Rate from Rx Misuse Overdose=

INTEG (Overdose Deaths from Misuse-Drop out Rx Misuse Deaths from Previous Month, Initial Number of People Misusing Rx Drugs *Risk of Overdose Death (ROD)*FR OD)

**Unit**: People

1. Susceptible=

INTEG (Quitting Illicit Drug Use + Quitting Prescription Misuse-Initiate Illicit Drugs-Initiate Misuse , "(N) Total Population"- Initial Number of People Misusing Rx Drugs - Initial Number of Illicit Drugs Users)

**Unit**: People

1. FR Switching=

SMOOTH (PULSE (start to switch , duration switch )*((Max FR Switching-Min FR Switching)*EXP(-Number of Opioid Analgesic Rx per 100 CT Resident per Month/Beta Coefficient3)), Time to switch)+Min FR Switching

**Unit**: 1/Month

1. Initiate Misuse=

Risk of Rx Misuse Initiation*Susceptible

**Unit**: People/Month

1. Total OD Illicit=

People who Use Illicit Drugs or Misuse Prescription Drugs *FR OD Illicit

**Unit**: People/Month

1. Quitting Illicit Drug Use=

People who Use Illicit Drugs or Misuse Prescription Drugs *Fraction Quitting Illicit Drug Use per Month

**Unit**: People/Month

1. Narcan Use=

"911 Calls and AMR Assistance with ODs"*(Total OD+Total OD Illicit)

**Unit**: People/Month

1. Switch to Illicit Drugs=

People who Misuse Prescription Drugs*FR Switching

**Unit**: People/Month

1. Total OD=

People who Misuse Prescription Drugs*FR OD

**Unit**: People/Month

1. Total Users=

110714

**Unit**: People

~ Total number of people who misuse prescription drugs or use illicit drugs in year 2009, based on SAMHSA reports

1. switch year=

IF THEN ELSE((Integer(Time/12)=Time/12), 1 , 0)

**Unit**: Dmnl

1. Total OD Yearly ED Visits=

SAMPLE IF TRUE ((Integer(Time/12)=Time/12), Total OD ED Visits , Total OD ED Visits)

**Unit**: People

1. Total Overdose Monthly Deaths 0=

SAMPLE IF TRUE( (Integer(Time)=Time):AND:(Time>0), Total Overdose Monthly Deaths , Initial Overdose Deaths)

**Unit**: People

1. Total Overdose Monthly Deaths=

Monthly Death Rate from Illicit Drug Overdose + Monthly Death Rate from Rx Misuse Overdose

**Unit**: People

1. switch every month=

IF THEN ELSE ((Integer (Time)=Time), 1 , 0)

**Unit**: Dmnl

1. Drop out Rx Misuse Deaths from Previous Month=

switch every month*Monthly Death Rate from Rx Misuse Overdose/TIME STEP

**Unit**: People/Month

1. Drop out Illicit Drug Deaths from Previous Month=

Monthly Death Rate from Illicit Drug Overdose*switch every month/TIME STEP

**Unit**: People/Month

1. Quitting Prescription Misuse=

People who Misuse Prescription Drugs*Fraction Quitting Rx Opioid Use per Month

**Unit**: People/Month

1. Risk of Rx Misuse Initiation=

1/(1+EXP(-Number of Opioid Analgesic Rx per 100 CT Resident per Month/Beta1+Initial X1))

**Unit**: 1/Month

**Section C:**

1. Net Change In the Perception of Drug Risk=

DELAY N( Total Overdose Monthly Deaths 0 , Average Time To Perceive Risk , Total Overdose Monthly Deaths 0 , n )

**Unit**: People/Month

1. Situational Awareness=

Net Change In the Perception of Drug Risk/Minimum Number Of Death To Get Noticed

**Unit**: Dmnl

1. n=

1.92855

**Unit**: Dmnl

1. Perception of Drug Risk=

INTEG (Net Change In the Perception of Drug Risk,0)

**Unit**: People

**Section D:**

1. Modified Fear for People with GSL Knowledge due to Rate of Drug Arrests=

WITH LOOKUP (Total Arrests/Max Arrests, ([(0,0)-(10,10)],(0.072,0.230769),(1,1) ))

**Unit**: Dmnl

~ ([(0,0)-(1,1)],(0,1),(0.741,0.072) ) The number 0.072 is the sum of following numbers (%4.1+%3.1) from the CT HIDTA CDC Report. The report shows from the 293 overdose calls in the past 6 months by 46 officers in Connecticut who responded to at least one overdose call, 12 of those calls (4.1%) resulted in the victim of an overdose being arrested. 9 of those calls (3.1%) resulted in a witness to an overdose being arrested. Also from another survey from the Connecticut Department of Public Health (CT DPH) and Central Connecticut State University’s (CCSU), on basic understanding of the GSL and the corresponding fear of calling 911, among 65 respondents who had some knowledge of the GSL, 15 (23.0769%) are afraid to call 911 in order to prevent an overdose.

1. "911 Calls and AMR Assistance with ODs"=

EXP(-Total Fear of calling 911/Beta Coefficient)*FR Narcan

**Unit**: Dmnl

1. Learning through Peers=

"(C) Contact Rate"*Probability of Learning GSL from Peers*"General Population w/o GSL Knowledge"*("Individuals with Knowledge of GSL & Naloxone Access & with Fear" + "Individuals with GSL Knowledge & w/o Fear")/"(N) Total Population"

**Unit**: People/Month

1. "Individuals with Knowledge of GSL & Naloxone Access & with Fear"=

INTEG (Learning through Peers-Behavioral Change, initial number of people with GSL Knowledge)

**Unit**: People

1. Behavioral Change=

(Target w/o Fear*Individuals with Knowledge of GSL & Naloxone Access & with Fear)/Time Delay in Behavioral Change

**Unit**: People/Month

1. Modified Fear for People without GSL Knowledge due to Rate of Drug Arrests =

WITH LOOKUP (Total Arrests, ([(0,0)-(1,1)],(0.072,0.581395),(1,1) ))

**Unit**: Dmnl

~ ([(0,0)-(1,1)],(0.072,0.581395),(1,1) )The number 0.072 is the sum of following numbers (%4.1+%3.1) from the CT HIDTA CDC Report. The report shows from the 293 overdose calls in the past 6 months by 46 officers in Connecticut who responded to at least one overdose call, 12 of those calls (4.1%) resulted in the victim of an overdose being arrested. 9 of those calls (3.1%) resulted in a witness to an overdose being arrested. Also from another survey from the Connecticut Department of Public Health (CT DPH) and Central Connecticut State University’s (CCSU), on basic understanding of the GSL and the corresponding fear of calling 911, among 43 respondents who did not know about the GSL, 25 (58.1395%) are afraid to call 911 in order to prevent an overdose.

1. Individuals with GSL Knowledge & w/o Fear=

INTEG (Behavioral Change, 0)

**Unit**: People

1. Target w/o Fear=

(1- Modified Fear for People with GSL Knowledge due to Rate of Drug Arrests")

**Unit**: Dmnl

1. Total Fear of calling 911=

(XIDZ( Individuals with Knowledge of GSL & Naloxone Access & with Fear , (Individuals with Knowledge of GSL & Naloxone Access & with Fear + Individuals with GSL Knowledge & w/o Fear), 0 )+ Modified Fear for People without GSL Knowledge due to Rate of Drug Arrests *Fraction without GSL Knowledge)

**Unit**: Dmnl

1. Probability of Learning GSL from Peers=

1/(1+EXP(-Situational Awareness/Beta+ Initial X))

**Unit**: Dmnl

1. Fraction without GSL Knowledge =

General Population w/o GSL Knowledge/(N) Total Population

**Unit**: Dmnl

1. (N) Total Population=

3040863

**Unit**: People

~ The size of the total population 12 years and older

1. General Population w/o GSL Knowledge=

INTEG (-Learning through Peers, "(N) Total Population")

**Unit**: People

**Section E:**

1. Min Arrests=

1.92667

**Unit**: Dmnl

1. Max Arrests=

4.13167

**Unit**: Dmnl

1. Total Arrests=

(Max Arrests-Min Arrests)*EXP(-("Law Enforcement Officers with Knowledge about GSL & Naloxone Access Laws"/Total CT Law Enforcement Officers)/Beta Coefficient1)+Min Arrests

**Unit**: Dmnl

1. "Law Enforcement Officers with Knowledge about GSL & Naloxone Access Laws"=

INTEG (Learning through Officer Peers, Initial number of officers with GSL Knowledge)

**Unit**: People

1. Learning through Officer Peers=

(C) Contact Rate Officers*Probability of Learning GSL from Peers*Law Enforcement Officers w/o GSL Knowledge*("Law Enforcement Officers with Knowledge about GSL & Naloxone Access Laws")/Total CT Law Enforcement Officers

**Unit**: People/Month

1. Knowledge of GSL for LE=

"Law Enforcement Officers with Knowledge about GSL & Naloxone Access Laws"/Total CT Law Enforcement Officers

**Unit**: Dmnl

1. Total CT Law Enforcement Officers=

6656

**Unit**: People

~ Connecticut has 92 municipal police departments. As of April 2011, they employed a total of 6,656 police officers Source: https://www.cga.ct.gov/2011/rpt/2011-R-0194.htm

1. Law Enforcement Officers w/o GSL Knowledge=

INTEG (-Learning through Officer Peers, Total CT Law Enforcement Officers)

**Unit**: People

**Section 2: Estimation and Calibration of Model Parameters**

While some of the model parameters are obtained from various existing datasets and are enlisted in section 1, there are no comprehensive data available for some parameters. In this situation, calibrating the model statistically to data would be a helpful method to estimate the unknown parameters. The final values along with confidence intervals are provided in Table S1.

**Table S1 – Calibrated Parameter Values**

| **Model Section** | **Parameter [Definition]** | **Value in the Model & Unit of Analysis** | **95% Confidence Interval** |
| --- | --- | --- | --- |
| A | Time to Change Opioid Rx Rate | 99.843 Month | 90.2333 - 110.99 |
|  | Fraction Opioid Rx Rate [Fractional Increase in Opioid Rx rate per 100 people per month] | 0.000165409 /Month | 1e-06 - 0.000404969 |
|  | Beta Coefficient2 [Parameter in the exponential function defining change in opioid Rx rate based on changes in situational awareness] | 0.0747029 | 0.0668404 - 0.0835155 |
|  | Min Opioid Rx Rate [Minimum Opioid Rx rate per 100 people per Month] | 0.1 /Month | 0.1 - 0.507258 |
|  | start to switch2 [Time that Opioid Rx rate per 100 people starts to drop] | 56.1225 Month | 53.2969 - 58.7344 |
| B | Beta1 [Value of logistic growth rate in the logistic function defining risk of Rx Misuse Initiation based on changes in opioid Rx rate] | 100 | 20.3587 -100 |
|  | Initial X1 [Value of sigmoid point in the logistic function defining risk of Rx Misuse Initiation based on changes in opioid Rx rate] | 9.99843 | 9.78496 - 10 |
|  | Risk of Illicit Drug Use Initiation | 0.00235833 / Month | 0.00189318 - 0.00280923 |
|  | Beta Coefficient3 [Parameter in the exponential function defining switching from Rx Misuse to Illicit Drug use based on changes in Opioid Rx Rate] | 1.86722 | 1.821 - 1.91709 |
|  | Max FR Switching [Maximum switching from Rx Misuse to Illicit Drug use] | 0.999971 | 0.928943 - 1 |
|  | Min FR Switching [Minimum switching from Rx Misuse to Illicit Drug use] | 1e-06 | 1e-06 - 0.000458695 |
|  | start to switch [Time that switching from Opioid Rx Misuse to Illicit Drug Use picks up due to drop in Opioid Rx Rate] | 36.2859 Month | 36 - 37.9219 |
|  | duration switch [Length of time that switching from Opioid Rx Misuse to Illicit Drug Use picks up due to drop in Opioid rx Rate] | 56.7265 Month | 48.2922 - 140 |
|  | Time to switch [Time delay in switching from Opioid Rx Misuse to Illicit Drug Use due to changes in Opioid rx Rate] | 33.3239 Month | 29.6806 - 37.2632 |
|  | FR NMUOP [Fraction of Total Drugs Users who are Misusing Rx] | 0.733176 | 0.733063 - 0.73329 |
|  | Target ROD from Illicit Drug [Risk of death during overdose from using illicit drugs] | 0.207212 | 0.202077 - 0.212459 |
|  | Target ROD from Rx Misuse [Risk of death during overdose from misusing Rx] | 1e-08 | 1e-08 - 0.0126548 |
|  | ED visit rate | 1.37808 /Month | 1.20956 - 1.54654 |
|  | Fraction Quitting Rx Opioid Use per Month | 0.002 /Month | 0.00150905 - 0.002 |
|  | Fraction Quitting Illicit Drug Use per Month | 0.000744068 /Month | 0.000317207 - 0.00118387 |
|  | FR OD [Fraction of People who Misuse Prescription Drugs who have an overdose per Month] | 0.00113309 /Month | 0.00113155 - 0.00113393 |
|  | FR OD Illicit [Fraction of People with Illicit Drug Use who have an overdose per Month] | 0.00367176 /Month | 0.00367021 - 0.00367331 |
|  | FR Narcan [Fraction of 911 Calls that conclude to Narcan Use] | 0.64118 /Month | 0.617575 - 0.664721 |
| C | n [Order of delay in changes in perception of drug risk based on changes in total overdose deaths per month] | 1.92855 | 1 - 4 |
|  | Minimum Number of Death To Get Noticed | 793.274 People | 751.027 - 839.597 |
|  | Average Time To Perceive Risk | 13.1131 Month | 12 - 17.7579 |
| D | Initial X [Value of sigmoid point in the logistic function defining probability of learning about GSL from Peers] | 4.52028 | 4.51782 - 4.52274 |
|  | Beta [Value of logistic growth rate in the logistic function defining probability of learning about GSL from Peers] | 1.14382 | 1.07417 - 1.2235 |
|  | Time Delay in Behavioral Change [Delay in shift in behavior after learning about GSL to overcome fear and call 911 during overdose incident] | 300 Month | 42.2082 - 300 |
|  | Beta Coefficient [Parameter in the exponential function defining number of 911 calls and AMR assistance with overdoses based on changes in fear and GSL knowledge] | 3.06265 | 2.8531 - 3.29478 |
|  | Beta Coefficient1 [Parameter in the exponential function defining rate of drug arrests based on changes in GSL knowledge among patrol officers] | 0.232842 | 0.152238 - 0.347888 |
|  | (C) Contact Rate [number of contacts per month among general public] | 9.85526 /Month | 9.83006 - 9.88044 |
|  | initial number of people with GSL knowledge | 87.1341 People | 84.7433 - 89.6037 |
| E | (C) Contact Rate Officers [number of contacts per month among patrol officers] | 2.9801 /Month | 2.95703 - 3.00382 |
|  | initial number of officers with GSL Knowledge | 383.605 People | 375.647 - 391.745 |
